# Delirium associated with moxifloxacinin a patient with toxic epidermal necrolysis due to methazolamide: a case report

**DOI:** 10.3389/fphar.2026.1803575

**Published:** 2026-04-15

**Authors:** Ming-Yi Ruan, Ze-Hu Liu

**Affiliations:** 1 Department of Pharmacology, Hangzhou Third People’s Hospital, Hangzhou, China; 2 Department of Dermatology, Hangzhou Third People’s Hospital, Hangzhou, China

**Keywords:** delirium, methazolamide, moxifloxacin, stevens-johnson syndrome, toxic epidermal necrolysis

## Abstract

**Background:**

Toxic epidermal necrolysis (TEN) is a potentially life-threatening adverse drug reaction characterized by extensive epidermal detachment and mucosal erosions. While delirium is a known complication in critically ill patients, its association with TEN remains poorly characterized.

**Case:**

We report a case of moxifloxacin-induced delirium with psychotic features in a patient who developed TEN secondary to methazolamide following orbital trauma. Physical examination revealed erythematous plaques with multiple flaccid bullae and widespread skin sloughing involving more than 40% of the total body surface area. The SCORTEN score was calculated as 2, corresponding to a predicted mortality rate of 12.1%. The patient was treated with intravenous immunoglobulin, systemic glucocorticosteroids, and supportive care. During hospitalization, she developed delirium attributable to moxifloxacin administration, which resolved promptly upon drug discontinuation.

**Conclusion:**

Delirium in critically ill dermatological conditions such as TEN is often multifactorial in origin. It may arise as a direct complication of severe systemic illness or as an adverse effect of necessary pharmacotherapy, with fluoroquinolones representing a well-recognized precipitant.

## 1 Introduction

Delirium is characterized by the acute onset of disturbances in attention, awareness, and cognition, with symptoms that tend to fluctuate over time (Thom et al., 2019). Its etiology is often multifactorial, with contributing factors including a proinflammatory state, severe pain, and ischemia—conditions commonly observed in patients with Stevens-Johnson syndrome (SJS) and toxic epidermal necrolysis (TEN). SJS and TEN are potentially life-threatening adverse drug reactions marked by extensive epidermal detachment and mucosal erosions (Bastuji-Garin et al., 1993; de Quintana-Sancho et al., 2016; Koh and Tay, 2009; Shah et al., 2024). Drugs have been implicated in over 75% of SJS/TEN cases (Ramien et al., 2022; Rufini et al., 2015; Sukasem et al., 2018), while *Mycoplasma* pneumoniae infection has also been identified as a causative agent, particularly in pediatric populations (Koh and Tay, 2009; Liu and Shen, 2019). In this report, we present a case of moxifloxacin-induced delirium with psychosis in a patient who developed TEN secondary to methazolamide use following orbital trauma.

### 1.1 Case description

A 44-year-old man presented to the emergency department with a 5-day history of general malaise and progressive, painful, widespread erythema accompanied by flaccid blisters. He also reported itchy and painful eyes, but denied dysphagia or dysuria. The patient had no prior history of allergic diseases and an unremarkable family history. However, he had sustained orbital trauma 1 month earlier, resulting in secondary glaucoma, for which he received oral methazolamide (25 mg twice daily) for 2 weeks. Methazolamide had been discontinued 2 weeks prior to presentation.

### 1.2 Physical examination upon admission

**FIGURE 1:**
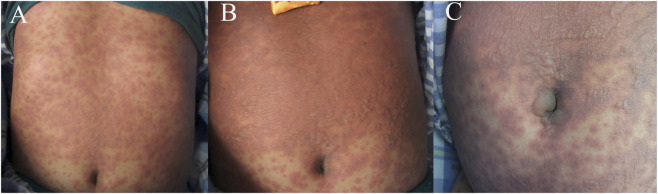
Clinical manifestations on day 0 **(A)**, day 1 **(B)** and day 2 **(C)** after admission.

Dermatological examination revealed multiple tender flaccid blisters, vesicles, and erythematous dusky-red plaques with erosion involving approximately 40% of the body surface area (Figure 1A). The Nikolsky sign was positive. Ophthalmologic evaluation showed eyelid edema and hyperemia of the bulbar conjunctiva and tarsal conjunctiva (both superior and inferior). Erosive lesions with detachment were also observed in the oral and genital mucosa. Vital signs on admission were significant for tachycardia (106 bpm) and intermittent fever ranging from 39.2 °C to 40 °C. The patient was alert and oriented at the time of examination.

### 1.3 Laboratory tests and imaging evaluation

Arterial blood gas analysis performed upon admission to the dermatology ward revealed: pH 7.458, PaCO_2_ 32.5 mmHg, PaO_2_ 75.4 mmHg, glucose 6.8 mmol/L, base excess −0.5 mmol/L, oxygen saturation 95.59%, total hemoglobin 13.5 g/dL, and lactate 3.1 mmol/L. Complete blood count was within normal limits, while high-sensitivity C-reactive protein (hsCRP) was elevated at 28.1 mg/L (normal 0–10 mg/L). Chest computed tomography was unremarkable. A skin biopsy was not performed due to patient refusal.

### 1.4 Diagnosis and treatment measures

Based on the recent history of methazolamide use and the characteristic clinical presentation with over 40% body surface area involvement, a diagnosis of methazolamide-induced toxic epidermal necrolysis (TEN) was established. The SCORTEN score on admission was 2, corresponding to a predicted mortality rate of 12.1%. Treatment was initiated immediately on day 0 with intravenous immunoglobulin (400 mg/kg daily for 5 days), intravenous methylprednisolone (80 mg daily), and supportive care.

**FIGURE 2:**
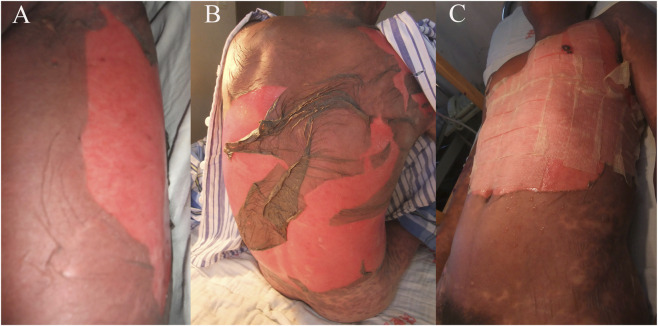
Clinical manifestations on day 3 **(A)**, day 4 **(B)** and day 5 **(C)** after admission.

**FIGURE 3:**
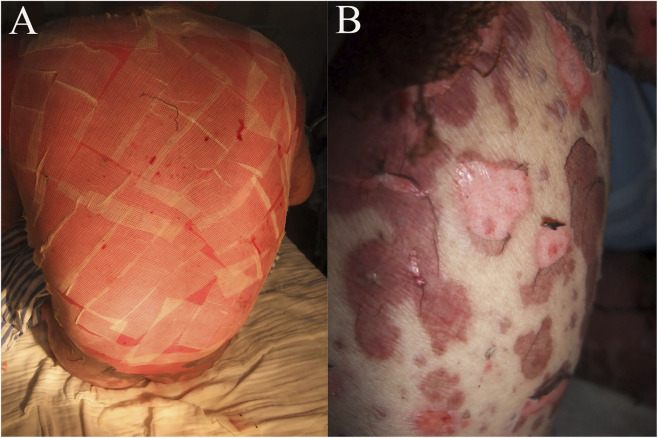
Clinical manifestations on day 6 **(A)** and day 13 **(B)** after admission.

Disease progression continued with extensive epidermal detachment through day 7 (Figures 1B,C, 2A–C, 3A). On day 8, the patient developed bacteremia due to methicillin-resistant *Staphylococcus haemolyticus* and was started on intravenous moxifloxacin and vancomycin. That same evening, he began experiencing visual hallucinations and became increasingly agitated. His wife reported acute confusion during the night, and the patient described vivid, well-defined, and colorful visual hallucinations, such as seeing “persons in the tree.” These episodes occurred primarily at night. Psychiatric consultation ruled out a personality disorder, and the patient remained coherent with intact memory during daytime hours. Moxifloxacin was suspected as the precipitant and was discontinued on hospital day 11, after which the delirium promptly resolved. By day 13, signs of re-epithelialization were observed (Figure 3B). The patient was discharged on day 20 in stable condition, with no need for further psychiatric follow-up.

## 2 Discussion

Delirium is the most common psychiatric syndrome observed in hospitalized patients, with an incidence of up to 87% among critically ill populations (Thom et al., 2019). Its etiology is often multifactorial, and the proinflammatory state, severe pain, and ischemia that accompany Stevens-Johnson syndrome (SJS) and toxic epidermal necrolysis (TEN) are likely contributing factors, although studies specifically linking delirium with these conditions remain scarce (Bidaki et al., 2017). One report describes a 61-year-old woman with heparin-induced SJS who developed worsening insomnia, persecutory delusions, and visual hallucinations (Bidaki et al., 2017). Another describes a 40-year-old woman with zonisamide-induced TEN who presented with delirium (Reinfeld et al., 2022). In the context of TEN, delirium may serve as a clinical biomarker of systemic disease severity and physiological instability.

Methazolamide, which contains a sulfone group analogous to that of sulfonamide drugs, has been associated with SJS/TEN, particularly in Korean and Chinese populations. A systematic review identified the HLA-B*5901 allele and the HLA-B*5901-Cw*0102 haplotype as significant risk factors for methazolamide-induced SJS/TEN in these populations (Tangamornsuksan and Lohitnavy, 2019). According to the ALDEN scoring system, methazolamide received a score of 3 (Table 1), indicating a possible association with SJS/TEN (Gronich et al., 2022; Sassolas et al., 2010).

**TABLE 1:** Details of the algorithm of drug causality for epidermal necrolysis (AlDEN).

Criterion			
Delay from initial drug component intake to onset of reaction (index day)	Values	Rules to apply	Score
Drug present in the body on index day	Suggestive +3	From 5 to 28 days	3
	Compatible +2	From 29 to 56 days	
	Likely +1	From 1 to 4 days	
	Unlikely −1	>56 Days	
	Excluded −3	Drug started on or after the index day	
	​	In case of previous reaction to the same drug, only changes for:Suggestive: +3: From 1 to 4 days Likely: +1: From 5 to 56 days	
Prechallenge/rechallenge	Definite 0	Drug continued up to index day or stopped at a time point less than five times the elimination half-life before the index day	−3
	Doubtful −1	Drug stopped at a time point prior to the index day by more than five times the elimination half-life but liver or kidney function alterations or suspected drug interactionsb are present	
	Excluded −3	Drug stopped at a time point prior to the index day by more than five times the elimination half-life, without liver or kidney function alterations or suspected drug interactions	
Dechallenge	Positive specific for disease and drug: 4	SJS/TEN after use of same drug	0
	Positive specific for disease or drug: 2	SJS/TEN after use of similar drug or other reaction with same drug	
	Positive unspecific: 1	Other reaction after use of similar drug	
	Not done/unknown: 0	No known previous exposure to this drug	
	Negative −2	Exposure to this drug without any reaction (before or after reaction)	
Type of drug (notoriety)	Neutral 0	Drug stopped (or unknown)	0
	Negative −2	Drug continued without harm	
Other cause	Strongly associated 3	Drug of the“high-risk”list according to previous case–control studies	3
	Associated 2	Drug with definite but lower risk according to previous case–control studies	
	Suspected 1	Several previous reports, ambiguous epidemiology results (drug“under surveillance”)	
	Unknown 0	All other drugs including newly released ones	
	Not suspected −1	No evidence of association from previous epidemiology studyd with sufficient number of exposed controlsc	
	​	Intermediate score = total of all previous criteria	​
Final score 3	Possible −1	Rank all drugs from highest to lowest intermediate score	​
	​	If at least one has an intermediate score >3, subtract 1 pointfrom the score of each of the other drugs taken by the patient (another cause is more likely)	​

<0, Very unlikely; 0–1, unlikely; 2–3, possible; 4–5, probable; ≥6, very probable.

ATC, anatomical therapeutic chemical; SJS, Stevens–Johnson syndrome; TEN, toxic epidermal necrolysis.

Previous research suggests that the incidence of fluoroquinolone-associated delirium or psychosis may be higher than previously estimated. In one veteran population, the incidence was 3.7%, compared to the 0.1% reported in post-marketing surveillance (Sellick et al., 2018). The exact mechanism by which fluoroquinolones induce delirium remains incompletely understood. Current evidence implicates direct pharmacological interference with GABAergic and glutamatergic neurotransmission, leading to an acute imbalance between cortical excitation and inhibition (Sellick et al., 2018). Moxifloxacin, in particular, exhibits good central nervous system (CNS) penetration and is frequently used in severely ill and vulnerable patients, which may explain its prominence in case reports and clinical practice (Tasleem and Viswanathan, 2011).

Medication-induced psychiatric symptoms often go unrecognized, particularly in critically ill patients where multiple confounding factors are present. In contrast, this case highlights a distinct, pharmacologically driven delirium temporally associated with moxifloxacin administration. The fluoroquinolone class—including moxifloxacin, levofloxacin, and ciprofloxacin—has a well-documented, mechanism-based risk of neurotoxicity.

The primary management priority in such cases remains the aggressive treatment of TEN and its systemic complications, including infection control, organ support, wound care, and multimodal analgesia and sedation, rather than the modification of adjunctive medications alone. In complex clinical scenarios such as TEN, where patients are critically ill and often receiving multiple high-risk medications and supportive therapies, a systematic approach is essential to identify the underlying causes of delirium. Clinicians should maintain a high index of suspicion for both disease-related and drug-induced etiologies. A detailed timeline correlating the onset and fluctuation of delirium symptoms with the initiation or adjustment of medications-as well as with objective markers of disease severity (e.g., SCORTEN scores, onset of sepsis, peak inflammation)-is crucial. The direct neurochemical interference mechanism is consistent with the clinical observation that delirium symptoms typically develop acutely within days of initiating moxifloxacin, with a median onset of 4 days, strongly supporting a drug-related cause. Discontinuing the suspected offending agent and observing for symptom resolution can serve both diagnostic and therapeutic purposes, as demonstrated in our patient, whose delirium resolved rapidly following moxifloxacin withdrawal. To our knowledge, this is the first case report of delirium possibly attributable to moxifloxacin in a patient with methazolamide-induced TEN. The patient did not received HLA typing to further confirm the association of methazolamide with TEN, which constitutes an important limitation of this study.

## 3 Conclusion

This case highlights the multifactorial nature of delirium in critically ill patients, particularly those with severe dermatological conditions such as toxic epidermal necrolysis (TEN). Delirium may arise either as a direct complication of the underlying severe skin disease or as an adverse effect of necessary pharmacotherapy, with fluoroquinolones representing a well-established precipitant. Thus, distinguishing between these etiologies is clinically essential, as the management approach differs fundamentally: disease-induced delirium requires aggressive treatment of the underlying condition, while drug-induced delirium necessitates prompt identification and discontinuation of the offending agent. This case underscores the importance of maintaining a high index of suspicion for medication-related neuropsychiatric symptoms in complex, critically ill patients including toxic epidermal necrolysis patients.

## Data Availability

The original contributions presented in the study are included in the article/supplementary material, further inquiries can be directed to the corresponding author.
